# Menstrual hygiene management practices and menstrual distress among adolescent secondary school girls: a questionnaire-based study in Nigeria

**DOI:** 10.4314/ahs.v22i2.46

**Published:** 2022-06

**Authors:** Ignatius O Nwimo, Nwamaka A Elom, Cajetan I Ilo, Uchechukwu A Ezugwu, Lazarus E Ezugwu, Ifeanyi J Nkwoka, Chukwuma P Igweagu, Chukwuma G Okeworo

**Affiliations:** 1 Department of Human Kinetics and Health Education, Ebonyi State University, Abakaliki, Nigeria; 2 Department of Medical Rehabilitation, University of Nigeria, Nsukka, Nigeria; 3 College of Health Technology, Oji-River, Enugu State, Nigeria; 4 Department of Community Medicine, Usmanu Danfodiyo University, Sokoto, Nigeria; 5 Department of Community Medicine, Enugu State University of Science and Technology, Enugu, Nigeria; 6 Department of Sociology and Anthropology, Nnamdi Azikiwe University, Awka, Nigeria

**Keywords:** Menstrual hygiene, management practices, menstrual distress, adolescent secondary school girls

## Abstract

**Background:**

Menstruation is a common and normal experience during the reproductive age of adolescent females and if not well managed might expose the female to menstrual distress.

**Aim & Objectives:**

To determine the menstrual hygiene management practices and menstrual distress among adolescent secondary school girls.

**Methods & Materials:**

Six hundred participants randomly drawn participated in the study. A structured questionnaire which had two inventories was used to collect data. The first inventory was a self-developed Menstrual Hygiene Management Practices Questionnaire (MHMPQ) (r = 0.71) and the second one was a modified 11-item Menstrual Distress Scale (MDS) (r = 0.74). Data were analyzed using descriptive and inferential statistics.

**Results:**

Results showed that menstrual hygiene management practices of the girls were poor and they were very highly distressed. Statistically significant differences were observed in menstrual hygiene management practices among the girls with regard to age, location of residence and occupation of mother and on menstrual distress in relation to occupation of father (p < 0.05). Significant positive relationship between menstrual hygiene management practices and menstrual distress was observed.

**Conclusion:**

The findings accentuate the need for a caring atmosphere for menstrual sanitation both at home and in school.

## Introduction

Menstruation is a common and normal experience during the reproductive age of the girl child. It is a natural process that occurs monthly in healthy adolescent or teenage girls. The first menstruation occurs between 11 and 15 years with a mean of 13 years^1^. It takes place during adolescent period in which dominant physiological and emotional changes take place. It is an essential period where females are preparing and adjusting themselves to manage their menstrual bleeding in safe and clean way^2^. This is also the ideal time that girls often join different environments including high schools and try to plan for their next adulthood life. However, most adolescent girls enter their puberty stage without preparing themselves due to the shortage of adequate information^3^.

Most women are uncomfortable to discuss issues regarding ‘menses’ as it has a social taboo and adolescent girls could not have access to gain adequate information^2^,^4^. More over the small information they get most regularly from spiritual institutions, peers and family associates is frequently discriminating and bordered by misperceptions^5^. For instance, people in the third world like Ethiopia often see menstruation as something occurring as an outcome of being cursed, a sign of diseases, and punishment from God, a lifelong process and others^5^, ^6^. As a consequence, teenage girls see menstruation as something discomforting that ought to be kept hidden^7^,^8^. This can amplify the susceptibility of adolescent girls to have mental, emotional and physical problems^9^. These conditions further impair the daily activities, academic performance, school attendance, and social relationships of adolescent girls^10^, ^11^.

Menstrual cleanliness is an issue that every girl and or woman has to deal with once she enters adolescence around the age of 11 years and until she reaches the menopause in her 40's. Normally, women spend roughly 2,100 days menstruating which is equal to about six years of their life^12^, ^13^. Menstruation is a-28 day happening that needs access to suitable materials and facilities, without which, the female suffers from poor menstrual hygiene which restricts her movement and self-confidence^14^ and in most instances may give rise to menstrual distress.

Menstrual distress is used as a group of negative symptoms which have been correlated with menstrual cycle pain and discomfort. The symptoms include pain, water retention, autonomic reactions, mental distress, impaired concentration, behavior change and arousal^15^, ^16^. Dysmenorrhea is the most familiar menstrual distress syndrome and one of the most common gynecologic problems in women of all ages. About 10% of women who have complained of dysmenorrhea have pain sufficiently strong to impede their performance for about 1–3 days in a calendar month. Even though some women can be discomforted several hours before onset of flow, symptoms typically begin with menstruation^17^, ^18^.

Globally, there are 1.2 billion young people between the ages of 10 and 19. Nearly 90% of them live in third world nations, and approximately 600 million are female^19^. Though teenage period is normally a time of good health, several developmental and social changes take place during this period, many of which have health implications, especially for girls. Mosof the hundreds of millions of girls in the developing world live in conditions and circumstances that make them vulnerable to health and social risks. These adolescent girls are often poor, out-of-school, married, migrants, members of ethnic minorities, or engaged in unsafe labor.

Adolescent girls consist of 20% of the total global population, of which 85% live in developing countries^20^. Preponderance of secondary school girls in Nigeria are adolescents. They represent a significant (44.8%) segment of the country's population^21^. Studies suggest adolescents, who also include those in Nigeria, face abundant challenges, which could be unfavorable to their health^22^,^23^. Such challenges might comprise those connected with cleanliness during menstruation.

Many studies have examined menstruation related distress among women with the aims of addressing poor perceptions of menstruation related to a lack of education and improving negative attitudes toward menstruation^24^–^25^. As the culture of the girl child plays important roles in shaping their menstrual experiences, the type and degree of menstrual distress vary widely, depending on the specific culture^26^, ^27^. Consequently, reports on menstrual distress in different nations have taken care to consider unique factors and to use their local dialect when assessing females to better understand the subtle nuances^28^, ^29^. In carrying out such studies, many authors have used already existing research tools, translated, and used in their studies.

Some of the commonly used instruments for menstrual symptoms include the Menstrual Distress Questionnaire^30^, Shortened Premenstrual Assessment Form31 and DSM-5-based symptom diary for diagnosing severe PMS and PMDD^32^.

Studies^12^, ^33^–^51^ over the years documented aspects of menstrual hygiene practices of adolescent girls in both developed and developing countries. Nevertheless, none of these studies measured age, occupation of father and mother and number of older sisters as significant variables that could positively or otherwise affect the menstrual hygiene practices of adolescent secondary school girls. The present study therefore was designed to ascertain the menstrual hygiene practice as it affects menstrual distress among adolescent secondary schools girls in Ebonyi State, Nigeria.

## Methods

### Participants and Sampling

Between August and September 2020, a cross-sectional survey was carried out among 600 secondary school adolescent girls randomly drawn through the multi-stage procedure. The first stage involved the selection of 2 out of 3 already existing clusters in the area under study in form of education zones. The second stage involved randomly drawing 6 secondary schools from each zone using the systematic simple random sampling technique. A list of secondary schools from each of 2 education zones selected in stage one facilitated the sampling. The third stage involved drawing 100 participants in each secondary school using convenient sampling approach of first to be sighted.

### Inclusion criteria

All adolescent secondary school girls within the age bracket of 10 to 19 years and must be in forth or fifth years (i.e., SS 1 or SS 2) in the secondary school were qualified to be included in the study.

### Exclusion criteria

Girls below 10 years and above 19 years were excluded in the study.

### Questionnaire

The questionnaire used in the data collection process was a 25-item instrument arranged in three sections; A, B and C. Section A, contained 6 items about the personal variables of the participants. Section B, consisted of 11 items of menstrual hygiene management statements in which the respondents were required to indicate on a 4-point scale, the level of agreement or disagreement on how they feel about the statements strongly agree (SA), Agree (A), Disagree (D) and Strongly disagree (SD). Section C, is a modified 8-item^52^, ^53^ menstrual distress scale in which the respondents were required to indicate on a 4-point scale, the frequency with which each item affects them during the period of menses using always (AL), sometimes (ST), rarely (RA) and never (NE).

Five experts in health education from two institutions of higher learning in Enugu State were used for validating the instrument. Thirty female adolescent secondary school students, in a secondary school not included in the study, were used for test of reliability. Each scale (menstrual hygiene management, Cronbach α = 0.71; menstrual distress, Cronbach α = 0.74) yielded reliability coefficient based on criterion of 0.60 acceptable for good instruments^54^.

### Variables

#### Outcome Variables

The menstrual hygiene management scale included 11 items which were scored using a 4-point scale (1 = strongly disagree to 4 = strongly agree) with higher scores indicating greater levels of good or healthy menstrual hygiene management. For analysis, these items were summed to create a continuous scale which could range from 0.0 to 4.0.

The menstrual distress scale included 8 main items with sub-items which were scored using a 4-point scale (1 = never to 4 = always) with higher scores indicating greater levels of menstrual distress. For analysis, these items were summed to create a continuous scale which could range from 0.0 to 4.0

#### Explanatory Variables

Explanatory variables included 6 personal variables including age, location of residence, class of study, occupation of father, occupation of mother and number of senior sisters.

### Procedure

The principal of each secondary school used in the study granted the researchers permission before data collection. A consent note with the explanation for the research purpose, method of response and assurance of anonymity was attached to each copy of the questionnaire Six hundred copies of the questionnaire were administered on the students during a school recreation period and were collected immediately after completion.

### Ethical Consideration

Ethical approval was given by Ebonyi State University Medical School Research Ethics Committee. A consent note was attached to each copy of the survey which participants read and agreed to participate in the study.

### Data Analysis

The completed copies of the questionnaire were examined for completeness of responses and copies that had incomplete responses were discarded. Out of 600 copies of the questionnaire administered; 521 representing about 86.8% return rate, were used for analysis. In describing the data, percentages, means and standard deviations were used to describe the respondents' demographic characteristics, menstrual hygiene management and menstrual distress, respectively. In describing menstrual hygiene management practice among the girls, a mean value of 2.5–4.0 implied good practice and a mean below 2.5 was adjudged poor practice. On the hand, in describing menstrual distress, a mean of 3.1–4.0 was adjudged that the girls were very highly distressed (VHD), 2.1–3.0 implied highly distressed (HD), 1.1–2.0 implied that the girls were lowly distressed (LD), and 0.1–1.0 implied that the girls were very lowly distressed (VLD).

Pearson's correlation was run to establish the relationship between menstrual hygiene management and menstrual distress among the participants. In order to describe the relationship between the variables, interpretation of the value of “r” was adopted55. In the explanation, a value of 0.01–0.19 was adjudged “very low” relationship; 0.20–0.39 “low”; 0.40–0.69 “moderate”; 0.70–0.89 “high”; 0.90–0.99 “very high”, and 1.0 “perfect” relationship. A plus (+) or (-) sign indicated whether the correlation was positive or negative. Stepwise multiple regression analysis was employed to verify the significance of the relationship between the variables.

T-test statistic was used to analyze data in order to ascertain whether the differences existing in menstrual hygiene management and menstrual distress of respondents in relation to location of residence and class of study were significant. On the hand, analysis of variance (ANOVA) was used to establish whether the differences found in the menstrual hygiene management and menstrual distress among the respondents in relation to age, occupation of father, occupation of mother and number of senior sisters were significant. An alpha level of 0.05 was set for the entire tests. All data analyses were done with IBM Statistical Package for Social Sciences Version 23.0 for Windows.

## Results

Data in Table 1 show the demographic characteristics of the respondents. The average age of the respondents is 16.3 years with majority (201, 38.6%) of them being 16 years old, more than half (266, 51.1%) living in the urban area and about half (263, 50.5%) are in senior secondary (SS 2). Majority parents (Father 320, 61.4%; Mother 288, 55.3%) are farmers and house wives, respectively. More than half (265, 50.9%) have one senior sister and 166(30.9%) have two senior sisters and more.

**Table 1:** Demographic Characteristics of Respondents (N = 521)

Variables	Number	Percentages
**Age**		

Less than 16 years	146	28.0
16 years	201	38.6
17 years	174	33.4

**Location of residence**		
Urban area	266	51.1
Rural area	255	48.9

**Class**		
SS 1	258	49.5
SS 2	263	50.5

**Occupation of father**		
Teaching/civil servant	98	18.8
Farming	320	61.4
Trading	77	14.8
Driving	26	5.0

**Occupation of mother**		
Teaching/civil servant	102	19.6
Farming	23	4.4
Trading	108	20.7
House wife	288	55.3

**Number of senior sisters**		
None	95	18.2
0ne	265	50.9
Two and above	161	30.9

Data in Table 2 show that most girls' mean menstrual hygiene management indices and cumulative MHM are below 2.50 indicating that their MHM was poor. Exceptions are ‘wiping from back to front following defecation (3.07±0.88) and clean sanitary pads/materials should be used since menstruation is dirty’ (2.54±0.54) that appear somehow good.

**Table 2:** Menstrual Hygiene Management (MHM) Practices

S/N	Statement		SD	Dec.
7.	Clean sanitary pads/materials should be used since menstruation is dirty	2.54	0.59	Good
8.	Sanitary pads/materials should be changed frequently	2.39	0.52	Poor
9.	Wiping from front to back following urination during menses	2.47	0.56	Poor
10.	Using highly absorbent pads during a time of light blood loss	2.46	0.57	Poor
11.	Use of pads when not menstruating (e.g., to absorb vaginal secretions)	2.42	0.57	Poor
12.	Wiping from back to front following defecation	3.07	0.88	Good
13.	Safe disposal of used menstrual materials or blood	2.47	0.55	Poor
14.	Douche (forcing liquid into the vagina)	2.29	0.59	Poor
15.	Hand-washing after changing a sanitary pad	2.47	0.59	Poor
16.	Having sex during menstruation	2.14	0.68	Poor
17.	Menstrual clothes should not be washed with other clothes	2.39	0.57	Poor
	**MHM**	2.48	0.24	Poor

Table 3 shows mean and standard deviation of each index of menstrual distress among the adolescent girls. Judging from the overall data, only in six of the indices that the girls are very highly distressed, indicating that in most of the indices the girls are highly, lowly or very lowly distressed. Specifically, the girls have issues with pain (2.51±0.35, highly distressed), concentration (2.24±0.31, highly distressed), behavioral change (2.09±0.38, lowly distressed), autonomic reaction (2.90±0.48, highly distressed), water retention (3.05±0.78, highly distressed), negative affect (3.02±0.67, highly distressed), arousal (2.86±0.65, highly distressed), and control (2.72±0.68, highly distressed).

**Table 3:** Menstrual Distress (MD)

Variables		SD	Dec.
Muscle stiffness (find it difficult to move, especially after rest)	2.43	0.53	HD
Headache	2.39	0.57	HD
Cramps (having muscle pain)	2.95	0.87	HD
Backache	2.37	0.59	HD
Fatigue (feeling tired)	2.39	0.54	HD
**Pain**	**2.51**	**0.35**	**HD**
Insomnia (finding it difficult to sleep)	2.32	0.62	HD
Forgetfulness	2.37	0.63	HD
Confusion	2.39	0.59	HD
Lowered judgment	2.43	0.55	HD
Difficulty concentrating	2.41	0.61	HD
Distractible	2.38	0.64	HD
Accidents	1.81	0.73	VLD
Lowered motor coordination	1.80	0.75	VLD
**Concentration**	**2.24**	**0.31**	**HD**
Lowered school or work performance	1.81	0.76	HD
Take naps; stay in bed	2.27	0.63	HD
Stay at home	2.06	0.78	HD
Avoid social activities	2.21	0.71	HD
Decreased efficiency	2.07	0.70	HD
**Behavioral Change**	**2.09**	**0.38**	**HD**
Dizziness, faintness	2.39	0.59	HD
Cold sweats	3.41	0.80	VHD
Nausea, vomiting	3.09	0.71	VHD
Hot flashes	2.73	1.04	HD
**Autonomic Reaction**	**2.90**	**0.48**	**HD**
Weight gain	3.45	0.77	HD
Skin disorders	2.64	1.09	HD
Painful breasts	3.23	0.95	VHD
Swelling	2.86	0.99	HD
**Water Retention**	**3.05**	**0.78**	**VHD**
Crying	3.39	0.84	VHD
Loneliness	3.11	1.05	VHD
Anxiety (feeling worried)	3.52	0.77	VHD
Restlessness (inability to rest)	2.99	1.09	HD
Irritability (easily getting annoyed)	2.80	1.17	HD
Mood swings (rapid changed in mood)	2.61	0.94	HD
Depression (feeling sad)	2.84	1.15	HD
Tension (feeling discomfort)	2.91	1.05	HD
**Negative Affect**	**3.02**	**0.67**	**VHD**
Affectionate	2.58	1.03	HD
Orderliness	2.98	0.91	HD
Excitement	2.99	0.89	HD
Feelings of well-being	3.01	0.92	VHD
Bursts of energy, activity	2.73	0.83	HD
**Arousal**	**2.86**	**0.65**	**HD**
Feeling of suffocation	2.80	0.95	HD
Chest pains	2.79	1.06	HD
Ringing in the ears	2.64	0.91	HD
Heart pounding	2.66	0.91	HD
Numbness, tingling (loss of sensation)	2.84	1.10	HD
Blind spots, fuzzy vision	2.59	0.85	HD
**Control**	**2.72**	**0.68**	**HD**

Table 4 shows the summary of t-Test and ANOVA analysis of menstrual hygiene management (MHM) practices and menstrual distress in relation to respondents' demographic variables. The data show that there are significant differences in MHM practices in relation to age (F = 3.47, p < 0.05), location of residence (t = 5.15, p < 0.05) and occupation of mother (F = 10.00, p < 0.05). In relation to MD, it is only in occupation of father (F = 4.32, p < 0.05) that a significant difference is observed.

**Table 4:** Summary of t-Test and ANOVA Analysis of Menstrual Hygiene Management (MHM) Practices and Menstrual Distress of Respondents' Demographic Variables

Variables	N			SD	Cal. value	p-value
**MHM**						
**Age**						
Less than 16 years	146		2.42	0.24		
16 years	201		2.48	0.26	F = 3.475*	0.032
17 years	174		2.49	0.23		
**Location of residence**						
Urban area	266		2.41	0.26	t = 5.145*	0.000
Rural area	255		2.52	0.22		
**Class**						
SS 1		258	2.48	0.23	t = 1.756	0.080
SS 2		263	2.45	0.26		
**Occupation of father**						
Farming	98		2.43	0.24		
Trading	320		2.46	0.25	F = 2.318	0.075
Driving	77		2.48	0.22		
	26		2.57	0.17		
**Occupation of mother**						
Teaching/civil servant	102		2.42	0.25		
Farming	23		2.53	0.23	F = 10.003*	0.000
Trading	108		2.38	0.27		
House wife	288		2.51	0.22		
**Number of senior sisters**					
None	95		2.50	0.27		
0ne	265		2.44	0.25	F = 2.307	0.101
Two and above	161		2.48	0.23		
**MD**						
**Age**						
Less than 16 years	146		2.67	0.31		
16 years	201		2.67	0.28	F = 0.244	0.784
17 years	174		2.69	0.27		
**Location of residence**						
Urban area	266		2.67	0.28	t = 0.608	0.543
Rural area	255		2.68	0.29		
**Class**						
SS 1		258	2.67	0.28	t = 0.008	0.994
SS 2		263	2.67	0.29		
**Occupation of father**						
Teaching/civil servant	98		2.68	0.28		
Farming	320		2.69	0.26	F = 4.317*	0.005
Trading	77		2.67	0.39		
Driving	26		2.48	0.19		
**Occupation of mother**						
Teaching/civil servant	102		2.66	0.26		
Farming	23		2.73	0.29	F = 0.353	0.787
Trading	108		2.68	0.26		
House wife	288		2.67	0.30		
**Number of senior sisters**						
None	95		2.67	0.29		
0ne	265		2.66	0.29	F = 0.708	0.493
Two and above	161		2.69	0.28		

* Significant at p < 0.05

Table 5 shows the summary of Pearson correlation of relationship between menstrual hygiene management practice and menstrual distress. The data show positive correlation coefficient scores that range from 0.02–0.41 indicating very low to low relationship. The relationship between menstrual hygiene management practice and specific indices (pain r = 0.35, concentration r = 0.41; behavioral change r = 0.31) are significant and relationship between menstrual hygiene management practice and overall menstrual distress is also significant.

**Table 5:** Summary of Pearson Correlation of Relationship between Menstrual Hygiene Management Practice and Menstrual Distress

Variables	MHM Practice
**Pain**	
Pearson Correlation	0.348*
Sig. (2-tailed)	0.000

**Concentration**	
Pearson Correlation	0.407*
Sig. (2-tailed)	0.000

**Behavioral Change**	
Pearson Correlation	0.307*
Sig. (2-tailed)	0.000

**Autonomic Reaction**	
Pearson Correlation	0.057
Sig. (2-tailed)	0.193

**Water Retention**	
Pearson Correlation	0.019
Sig. (2-tailed)	0.662

**Negative Affect**	
Pearson Correlation	0.016
Sig. (2-tailed)	0.718

**Arousal **	0.055
Pearson Correlation	0.210
Sig. (2-tailed)	

**Control**	
Pearson Correlation	0.031
Sig. (2-tailed)	0.476

**MD**	0.158*
Pearson Correlation	0.000
Sig. (2-tailed)	

* Significant at p < 0.05

Table 6 shows that the multiple regression (R) value for ‘use of pads when not menstruating’ (e.g., to absorb vaginal secretions) is 0.09 shows a positive relationship, while the F-value = 3.99. The p-value is less than p = 0.05 indicating that there is a significant relationship between the variable and MHM practice and MD of the subjects. The value of regression weight β = 0.04, which accounts for 4.4% variance in the model indicates a low predictive value of MD among the adolescents. Not douching or not forcing liquid into the vagina follows a same trend but tends to contribute less variance (4.3%) in the model. When MHM is considered as a unit, it tends to 18.5% variance in the model indicating a higher but also low predictive value of MD among the adolescents. The relationship between other indices of MHM practice and MD of the subjects is not significant showing that individual indices of MHM do not predict MD.

**Table 6:** Summary of Stepwise Multiple Regression Analysis of Menstrual Hygiene Management (MHM) Practices and Menstrual Distress

Variables	R	R^2^	α	F-value	p-value
Clean sanitary pads/materials should be used since menstruation is dirty	0.074	0.005	0.036	2.854	0.092
Sanitary pads/materials should be changed frequently	0.021	0.000	0.011	0.221	0.638
Wiping from front to back following urination during menses	0.079	0.006	0.041	3.301	0.070
Using highly absorbent pads during a time of light blood loss	0.046	0.002	0.023	1.118	0.291
Use of pads when not menstruating (e.g., to absorb vaginal secretions)	0.087	0.008	0.044	3.987*	0.046
Wiping from back to front following defecation	0.045	0.002	0.015	1.070	0.302
Safe disposal of used menstrual materials or blood	0.068	0.005	0.036	2.419	0.120
Do not douche (not forcing liquid into the vagina)	0.090	0.008	0.043	4.232*	0.040
Hand-washing after changing a sanitary pad	0.079	0.006	0.038	3.301	0.070
Not having sex during menstruation	0.054	0.003	0.023	1.508	0.220
Menstrual clothes should not be washed with other clothes	0.063	0.004	0.032	2.087	0.149
a. **MHM (Predictor)**	0.158	0.025	0.185	13.327*	0.000
b. **Dependent variable (Menstrual Distress)**					

* Significant at p < 0.05

## Discussion

Data on demographic characteristics of the respondents in Table showed that the majority (38.6%) of respondents were aged 16 years old. This number was not amazing because, these days in Nigeria most children start going to school earlier than before, corroborating the findings of a previous study^56^. More than half (51.1%) the number of respondents lived in the urban area contrary to the observation of previous research^56^ and about half (50.5%) were in senior secondary (SS 2) and was expected. A good number of parents (Father 61.4%; Mother 55.3%) are farmers and house wives, respectively. The findings were expected because very good numbers of males in the area of study are farmers and most women are house wives. More than half (50.9%) had one senior sister and 166(30.9%) had two senior sisters and more contrary to the reports of previous research^56^.

Results in Table 2 revealed that menstrual hygiene management practice of the adolescent secondary school girls was poor. One probable reason for the poor menstrual hygiene management practice among the adolescents could be the dearth of significant others such senior sisters who could provide veritable information on the subject matter. A close look at the findings could show that most of the adolescent girls had one senior sister who may not have acquired enough information with which to assist the adolescents in matters relating the menstrual hygiene. However, there were some exceptions in the indices of the practice where the girls' MHM was good. These were in the practice of ‘wiping from back to front following defecation (3.07±0.88) and using clean sanitary pads/materials should be used since menstruation is dirty (2.54±0.54)’. The girls' MHM practice was expected to be better than what was observed because other simple activities capable of instilling healthy reproductive health life among the girls were poorly practiced. The poor practice may expose the girls to various reproductive health problems ranging from simple virginal odor that could be easily managed to complex bacterial vaginosis (BV) that has been cited to be more common in women with unhygienic menstrual management practices. The MHM practice may also lead to bad odor of menstrual blood which may put the girls at risk of being stigmatized^12^, ^37^. The above findings of the study corroborated findings did not observe a cheering MHM practice among girls studied some couple of years ago confirming the status of menstrual hygiene among the girls and very few girls used sanitary pad and others used pieces of cloths, faced problem of washing and drying^46^. However, the findings tended to be a variance with those of other researchers^43^. The variation in findings of the present study and that of the previous may have been as a result of level of development where the present study was conducted in a more or less a less developed area compared to the area where the previous one was carried out.

Results in Tables 3 showed that the girls suffered from menstrual distress albeit in varying dimensions, ranging from very highly to very lowly distress. It could be observed that the girls were distressed in issues related to pain, concentration, autonomic reaction, water retention, negative affect, arousal and control. It may be understood why the girls reported suffering from menstrual distress based on the poor nature of their menstrual hygiene. Common logic would have it that the poorer their MHM the more they are likely to suffer from menstrual distress. The results of the present study seem to be in line with those of previous studies^48^–^50^ that reported moderate to low menstrual distress among the girls the studied. The present study corroborating the findings of previous may not be far from similar characteristic shared among adolescent girls globally which might suggest global reproductive health education program that focus on adolescent girls.

It could be seen in Table 4 that there are significant differences in MHM practices in relation to age (F = 3.48, p < 0.05), location of residence (t = 5.15, p < 0.05) and occupation of mother (F = 10.00, p < 0.05). Similarly, relation to MD only in occupation of father (F = 4.32, p < 0.05) that a significant difference was observed. It was not disgusting observing differences in MHM practices of the girls with regard to their age and location of residence. This is because adolescent girls, no matter, share the same characteristics age could conferee some level of experience in matters relating to MHM practices. In relation to area of residence, adolescent girls residing in urban cities have the propensity to possess more and richer information than those residing the rural areas, therefore the difference observed in their MHM practices was not surprising. In most instances, occupation of the mother plays significant roles in changing the behavior of adolescent girls. That is to say that some occupations (e.g., teaching, house wife) might make the mother stay close to the girl child and provide information that might make a difference in her life. Therefore, the difference observed in the MHM practices in relation to occupation of the mother was not a surprise. The implication of the differences is the mitigation of the variables that have low MHM practices scores in order to scale up their practice. The results of the present seem to be in line with those of previous studies^51^, ^56^ who reported differences in MHM practices in relation to age and area of residence of the young girls the studies.

In relation to MD only in occupation of father (F = 4.32, p < 0.05) that a significant difference was observed. This was a surprising revelation that father's occupation could influence menstrual distress of girl child. MD is a function of poor menstrual hygiene as a results father's occupation should have been silent over the MD of the girl child. Though there is paucity of information on father's occupation as it relates to MD of the girl child, the present study could bridge the gap on this area. However, it has been suggested that men should get involved in community menstrual hygiene management58. When this suggestion becomes effective in any community, including Nigeria, the situation might change.

Results on the Pearson correlation of relationship between menstrual hygiene management practice and menstrual distress in Table 5 showed positive correlation coefficient scores (range 0.02–0.41) that indicate very low to low relationship. The relationship between menstrual hygiene management practice and specific indices (pain r = 0.35, concentration r = 0.407; behavioral change r = 0.31) and overall menstrual distress were significant which could be an indication that MHM practices had the potentials for predicting MD. It was not a surprise to observe the significant relationship existed between MHM and MD because common sense might have it that a rough road in life might end up with enough trouble to the individual. This may be the reason why it was insinuated that hygiene-related practices of women during menstruation are of considerable importance, as unhygienic practices have a health impact in terms of increased vulnerability to reproductive tract infections (RTI)^21^.

Results of multiple regressions in Table 6 showed that MHM contributed 18.5% variance in the model indicating low predictive value of MD among the adolescents. The relationship between individual indices of MHM practices and MD of the subjects were not significant showing that individual indices of MHM tended not predicting MD. These tend to validate the results of the Pearson's correlation in Table 5. The findings are in line with those of previous studies^2^, ^3^, ^5^, ^49^, ^59^.

## Conclusions and Recommendations

The finings of the study showed a good number of adolescent girls who participated in the study were 16 years old. Our study reported that the MHM practices of the girls were not cheering and that was the reason why they suffered recognizable MD. Menstrual hygiene practices are imperative at all period; the need for more cautious focus to special hygiene prior to and all through menses might add to an adolescent girl's comfort and self-confidence.

In our study it was observed there were significant differences in MHM practices among the adolescent girls in relation to demographic variables such as age, location of residence and occupation of mother. In relation to MD, it is only in occupation of father that a significant difference was reported. There was a positive and significant relationship between menstrual hygiene management practice and menstrual distress indicating that MHM practice could predict the occurrence of menstrual distress. We recommend that men should get involved in community menstrual hygiene management as has been suggested^58^. If this suggestion becomes effective in any community, including Nigeria, the situation might change. Menstrual hygiene should be associated to the hygiene teaching programme in all levels of education active involvement of both male and female teachers. Caring atmosphere for menstrual sanitation has to be provided both at home and in school. The findings of this research may not be used in making generalizations regarding other resident groups in Nigeria and in another place; who may be different to a great degree in social and economic circumstances. The adolescent girls studied may represent a significant cluster of the Nigerian inhabitants; therefore, information generated might be useful in the planning of prospective facsimile health-related programs on menstrual hygiene in all levels of education in Nigeria and other sub-Saharan Africa countries that may have same cultural resemblance with Ebonyi state.
